# Hydrophobic Alpha-Helical Short Peptides in Overlapping Reading Frames of the Coronavirus Genome

**DOI:** 10.3390/pathogens11080877

**Published:** 2022-08-03

**Authors:** Takashi Okura, Kazuya Shirato, Masatoshi Kakizaki, Satoko Sugimoto, Shutoku Matsuyama, Tomohisa Tanaka, Yohei Kume, Mina Chishiki, Takashi Ono, Kohji Moriishi, Masashi Sonoyama, Mitsuaki Hosoya, Koichi Hashimoto, Katsumi Maenaka, Makoto Takeda

**Affiliations:** 1 Department of Virology 3, National Institute of Infectious Diseases, Musashimurayama 208-0011, Tokyo, Japan; t-okura@niid.go.jp (T.O.); shirato@niid.go.jp (K.S.); kakizaki@niid.go.jp (M.K.); ssugimo@niid.go.jp (S.S.); 2 Management Department of Biosafety, Laboratory Animal, and Pathogen Bank, National Institute of Infectious Diseases, Musashimurayama 208-0011, Tokyo, Japan; 3 Center for Influenza and Respiratory Virus Research, National Institute of Infectious Diseases, Musashimurayama 208-0011, Tokyo, Japan; matuyama@niid.go.jp; 4 Department of Microbiology, Faculty of Medicine, Graduate Faculty of Interdisciplinary Research, University of Yamanashi, Chuo 409-3898, Yamanashi, Japan; tomohisat@yamanashi.ac.jp (T.T.); kmoriishi@yamanashi.ac.jp (K.M.); 5 Department of Pediatrics, School of Medicine, Fukushima Medical University, Fukushima 960-1295, Fukushima, Japan; kumetti@fmu.ac.jp (Y.K.); chishiki@fmu.ac.jp (M.C.); takashi5@fmu.ac.jp (T.O.); mhosoya@fmu.ac.jp (M.H.); don@fmu.ac.jp (K.H.); 6 Center for Life Science Research, University of Yamanashi, Chuo 409-3898, Yamanashi, Japan; 7 Division of Hepatitis Virology, Institute for Genetic Medicine, Hokkaido University, Sapporo 060-0808, Hokkaido, Japan; 8 Division of Molecular Science, Graduate School of Science and Technology, Gunma University, Kiryu 376-8515, Gunma, Japan; sonoyama@gunma-u.ac.jp; 9 Gunma University Center for Food Science and Wellness (GUCFW), Gunma University, Kiryu 376-8515, Gunma, Japan; 10 Gunma University Initiative for Advanced Research (GIAR), Gunma University, Kiryu 376-8515, Gunma, Japan; 11 Laboratory of Biomolecular Science, Faculty of Pharmaceutical Sciences, Hokkaido University, Sapporo 060-0812, Hokkaido, Japan; maenaka@pharm.hokudai.ac.jp; 12 Center for Research and Education on Drug Discovery, Hokkaido University, Sapporo 060-0812, Hokkaido, Japan; 13 Global Station for Biosurfaces and Drug Discovery, Faculty of Pharmaceutical Sciences, Hokkaido University, Sapporo 060-0812, Hokkaido, Japan

**Keywords:** coronavirus, SARS-CoV-2, overlapping frame, sub-genomic RNA, nidovirus, alpha-helix

## Abstract

In this study, we show that the coronavirus (CoV) genome may encode many functional hydrophobic alpha-helical peptides (HAHPs) in overlapping reading frames of major coronaviral proteins throughout the entire viral genome. These HAHPs can theoretically be expressed from non-canonical sub-genomic (sg)RNAs that are synthesized in substantial amounts in infected cells. We selected and analyzed five and six HAHPs encoded in the S gene regions of severe acute respiratory syndrome coronavirus 2 (SARS-CoV-2) and Middle East respiratory syndrome coronavirus (MERS-CoV), respectively. Two and three HAHPs derived from SARS-CoV-2 and MERS-CoV, respectively, specifically interacted with both the SARS-CoV-2 and MERS-CoV S proteins and inhibited their membrane fusion activity. Furthermore, one of the SARS-CoV-2 HAHPs specifically inhibited viral RNA synthesis by accumulating at the site of viral RNA synthesis. Our data show that a group of HAHPs in the coronaviral genome potentially has a regulatory role in viral propagation.

## 1. Introduction

The family *Coronaviridae* in the order *Nidovirales* includes significant human pathogens and animal viruses with diverse pathogenicity [1,2]. A novel coronavirus, severe acute respiratory syndrome coronavirus 2 (SARS-CoV-2) [3,4], is currently causing a pandemic and wreaking havoc on human society. Besides SARS-CoV-2, we have experienced two other severe respiratory disease outbreaks, one caused by SARS-CoV in 2002–2003 and one cause by Middle East respiratory syndrome coronavirus (MERS-CoV) in 2012 [5]. Infections caused by MERS-CoV have continued unabated, mainly in the Middle East region [6]. It has become evident that many animal (especially bat) coronaviruses closely related to these three emerging coronaviruses exist in nature [7,8,9,10,11,12]. Therefore, coronaviruses are expected to continue to be a threat. Other coronaviruses that infect humans (human coronaviruses (HCoVs)) include HCoV-229E, -NL63, -OC43, and -HKU1, which are responsible mainly for seasonal colds in children, but they can also cause lower respiratory infections in many cases [13]. Each coronavirus species has a distinct host range, and many animal coronaviruses also exist and cause severe diseases in their host animals [1,2]. Several animal coronaviruses are significant pathogens in the livestock and poultry industries [1,2]. Understanding coronaviruses is therefore becoming increasingly important.

The major coronaviral proteins are translated from sub-genomic (sg)RNAs synthesized by template switching using transcription-regulating sequences (TRSs). In addition, many non-canonical sgRNAs of unknown importance are also synthesized by TRS-independent mechanisms, and template switching occurs throughout the genome [14,15,16,17,18]. The amount of each non-canonical sgRNA is so small as to be considered meaningless, but the total amount is nearly one-third to one-half that of canonical sgRNAs [16,18,19]. These non-canonical sgRNAs can encode N-terminal truncated major proteins, but they can also encode proteins (or peptides) with entirely different amino acid sequences from the major viral proteins using frameshifted overlapping reading frames [14,20]. The encoding of different proteins in the same gene region by changing the reading frame is one of the gene expression strategies of coronaviruses [21,22,23,24]. This study found that coronaviruses potentially encode many hydrophobic short peptides with alpha-helix structures in the entire virus genome of approximately 30,000 bases in length and showed their unique properties. The data obtained in this study indicate that hydrophobic alpha-helical peptides (HAHPs) have the ability to co-regulate coronavirus-specific membrane fusion by spike proteins and viral RNA synthesis at the replication site, suggesting that coronavirus replication may be highly regulated by the expression of HAHPs. These data are also valuable information for the development of novel antiviral agents or inhibitors of viral replication using HAHPs or their analogous compounds.

## 2. Results

### 2.1. The Highly Biased Proportion of Hydrophobic Amino Acids in the Overlapping Reading Frame of the Coronavirus Genome

**Figure 1:**
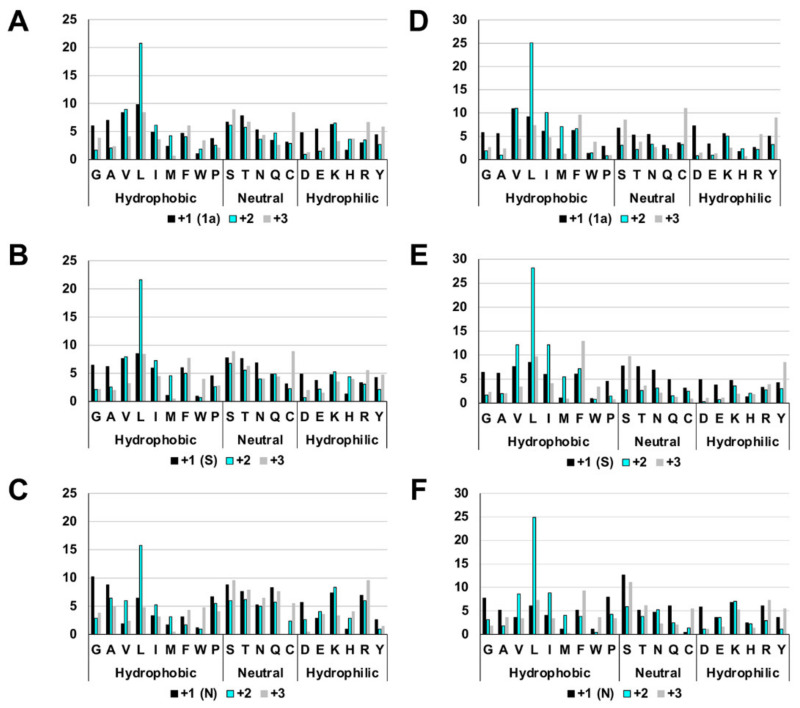
Amino acid compositions in different reading frames. (**A**) SARS-CoV-2 1a region. (**B**) SARS-CoV-2 S region. (**C**) SARS-CoV-2 N region. (**D**) HCoV-HKU1 1a region. (**E**) HCoV-HKU1 S region. (**F**) HCoV-HKU1 N region.

In this section, the frame encoding a major coronaviral protein is referred to as the +1 frame, and frames shifted back one base or two bases are referred to as +2 or +3 frames, respectively. The deduced amino acids in these frames revealed the existence of a highly biased proportion of hydrophobic amino acids in the +2 frame of coronaviruses. This feature was observed throughout the entire coronaviral genome, but it was somewhat challenging to assess unambiguously in the 3’-terminal region after the S gene because of the various accessory genes encoded there. An example of this is shown in Figure 1, which depicts the data for the 1a, S, and N gene regions of HCoV-HKU1 (GenBank accession: LC315651) and SARS-CoV-2 (GenBank accession: MN908947.3). In the +2 frame, the overall percentage of hydrophobic amino acids was high, but the percentage of leucine, in particular, stands out. Approximately 20–25% of the amino acids (i.e., one in four or five) were leucines. The proportion of charged hydrophilic amino acids was also significantly lower in the +2 frame. In addition, the number for methionine was markedly higher in the +2 frame.

**Figure 2:**
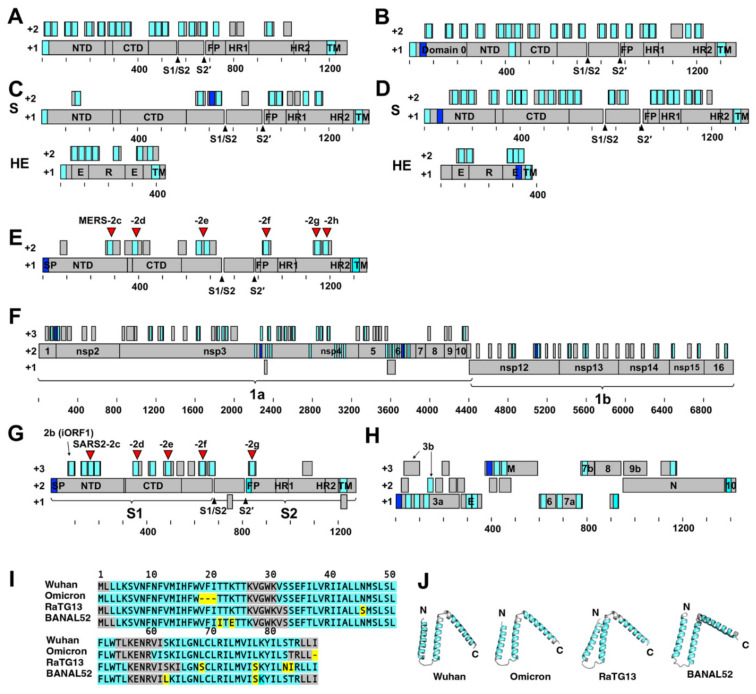
Schematic diagram of coronavirus genes and hydrophobic alpha-helical peptides (HAHPs). (**A**) HCoV-229E (GenBank accession LC654445). (**B**) HCoV-NL63 (LC654455). (**C**) HCoV-OC43 (LC654454). (**D**) HCoV-HKU1 (LC315651). (**E**) MERS-CoV (NC_019843) (**A**–**E**) Gray boxes in the +2 frames show all ORFs with ≥20 amino acids. Cyan and blue boxes show the primary and secondary, respectively, transmembrane alpha-helices predicted by SOSUI [25,26]. Only frames +1 (for major viral proteins) and +2 are shown. (**E**) The red inverted triangles indicate the HAHPs analyzed in this study. (**F**–**H**) SARS-CoV-2 genes. GenBank accession MN908947.3. Gray boxes in the overlapping frames show all ORFs with ≥20 amino acids. Cyan and blue boxes show the primary and secondary, respectively, transmembrane alpha-helices predicted by SOSUI [25,26]. (**F**) 1ab gene. (**G**) S gene. The red inverted triangles indicate the HAHPs analyzed in this study. (**H**) 3ab, E, M, 6, 7ab, 8, N, 9b, 10. (**I**) Amino acid sequence of the 2c peptide of SARS-CoV-2 (Wuhan and Omicron) and related bat coronaviruses (RaTG13 and BANAL52). Cyan shows the predicted transmembrane alpha-helices, and yellow shows the substitutions. (**J**) 2c structures predicted by Alphafold2.

This highly biased amino acid composition resulted in many short open reading frames (ORFs) with large amounts of hydrophobic amino acids in the +2 frame. Examples of this are shown in Figure 2A–H and Dataset S1–S7, which depict data from four HCoVs and three recently emerged severe respiratory coronaviruses. SOSUI 1.11 [25,26] and TMHMM 2.0 programs revealed that the deduced peptides in a majority of these ORFs (>20 amino acids in length) in the +2 frame are hydrophobic alpha-helical peptides or contain hydrophobic alpha-helical regions (Figure 2A–H, Dataset S1–S7). This feature was also common to animal coronaviruses (such as mouse hepatitis virus, porcine epidemic diarrhea virus, bovine coronavirus, infectious bronchitis virus, feline infectious peritonitis virus, and canine coronavirus) and SARS-CoV-, MERS-CoV-, and SARS-CoV-2-related bat or pangolin coronaviruses (such as RaTG13, BANAL52, BANAL103, BANAL236, RShSTT182, NeoCoV, and Rc-o319) [9,10,11,12,27]. These peptides are referred to collectively in this paper as hydrophobic alpha-helical peptides (HAHPs).

### 2.2. Membrane Fusion Inhibitory Activity of SARS-CoV-2 HAHPs

**Figure 3:**
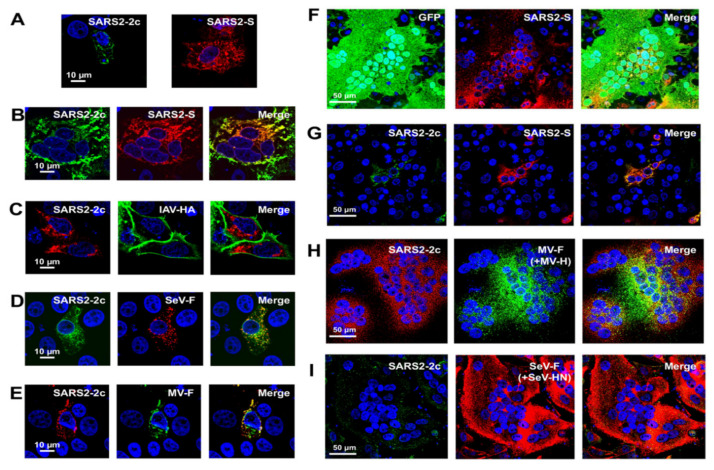
Intracellular localization and membrane fusion inhibitory activity of SARS-CoV-2-2c. VeroE6/TMPRSS2 cells were singly transfected with a plasmid expressing SARS-CoV-2 (SARS2)-2c or SARS2-S (**A**) or were co-transfected with expression plasmids for a combination of SARS2-2c plus SARS2-S (**B**) or SARS2-2c plus influenza A virus (IAV) HA (**C**). HeLa/TMPRSS2 cells or Vero/SLAM cells were co-transfected with expression plasmids for a combination of SARS2-2c plus Sendai virus (SeV) F (**D**) or SARS2-2c plus measles virus (MV) F (**E**). For the membrane fusion inhibitory activity assays, the VeroE6/TMPRSS2, Vero/SLAM, or HeLa/TMPRSS2 cells were co-transfected with expression plasmids for a combination of SARS2-S plus GFP (**F**) or SARS2-2c (**G**), MV F and H plus 2c (**H**), or SeV F and HN plus SARS2-2c (**I**). At 48 hpt, the cells were fixed and stained with primary antibodies (anti-HA tag Ab for SARS2-2c, anti-SARS2-S Ab, mAb12-1G6 for IAV HA, anti-SeV F Ab, or anti-MV F Ab). Nuclei were stained with DAPI.

**Figure 4:**
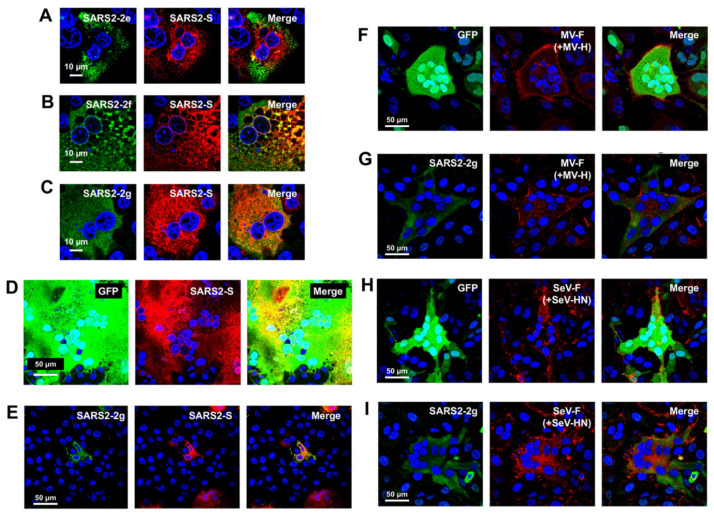
Intracellular localization and membrane fusion inhibitory activity of SARS-CoV-2-2d, -2f, and -2g. VeroE6/TMPRSS2 cells were co-transfected with expression plasmids for a combination of SARS-CoV-2 (SARS2)-2d (**A**), -2f (**B**), or -2g (**C**) plus SARS2-S. For the membrane fusion inhibitory activity assays, VeroE6/TMPRSS2, Vero/SLAM, or HeLa/TMPRSS2 cells were co-transfected with expression plasmids for a combination of SARS2-S plus GFP (**D**) or SARS2-2g (**E**), MV F and H plus GFP (**F**) or 2g (**G**), or SeV F and HN plus GFP (**H**) or SARS2-2g (**I**). At 48 hpt, the cells were fixed and stained with primary antibodies (anti-HA tag Ab for SARS2-2d, -2f, and -2g, anti-SARS2-S, anti-MV F, or anti-SeV F Abs). Nuclei were stained with DAPI.

This study first focused on the five HAHPs within the S gene region of SARS-CoV-2 (GenBank accession: MN908947.3) because non-canonical sgRNAs that can likely express these HAHPs have been detected in infected cells, albeit in minimal amounts [14]. In this paper, these are referred to as SARS2-2c, -2d, -2e, -2f, and -2g (Figure 2G, Dataset S5). Among them, the longest was SARS2-2c, consisting of 87 amino acids, and this ORF was conserved in closely related bat coronavirus genomes (Figure 2I). In addition, SOSUI 1.11 [25,26], TMHMM 2.0, and Alphafold2 [28] predicted that SARS2-2c has three transmembrane alpha-helices (Figure 2I,J). When SARS2-2c was expressed alone in cells, it was distributed near the cell periphery, suggesting its binding to or incorporation into the plasma membrane (Figure 3A). Furthermore, when expressed together, SARS2-2c fully co-localized with the S protein of SARS-CoV-2 (SARS2-S) (Figure 3B). The co-localization was specific to SARS2-S protein because SARS2-2c co-localized poorly with other viral glycoproteins (e.g., hemagglutinin of influenza A virus and the fusion proteins of Sendai and measles viruses) (Figure 3B–E). Notably, the SARS2-2c inhibited syncytium formation by SARS2-S protein but not by measles virus or Sendai virus glycoproteins (Figure 3F–I). SARS2S-2c inhibited syncytium formation by the S protein of all tested SARS-CoV-2 variants (Alpha, Beta, Gamma, Delta, and Omicron) (Supplementary Figure S1). Similar analyses were performed for four other short HAHPs (SARS2-2d, -2e, -2f, and -2g) of 29–39 amino acids in length. Among them, SARS2-2g, which partially co-localized with SARS2-S (Figure 4C), inhibited SARS2-S-mediated syncytium formation (Figure 4D,E). The SARS2-2d function was unclear because the expression of SARS2-2d was not able to be confirmed by immunofluorescence assay (Figure 4A–C). This inhibition was also specific to SARS2-S protein because SARS2-2g did not inhibit the syncytium formation mediated by measles virus or Sendai virus glycoproteins (Figure 4F–I).

### 2.3. Membrane Fusion Inhibitory Activity of MERS-CoV HAHPs

**Figure 5:**
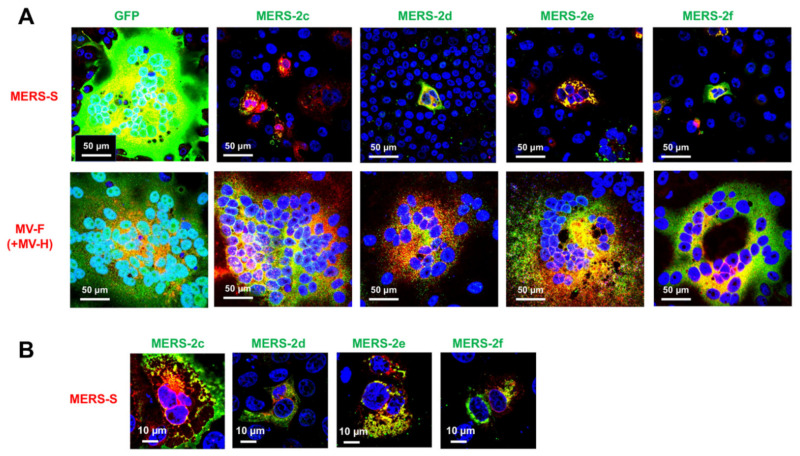
Membrane fusion inhibitory activity of MERS-CoV-2c, -2d, -2e, and -2f. VeroE6/TMPRSS2 or Vero/SLAM were co-transfected with expression plasmids for a combination of MERS-S or MV F and H plus GFP, MERS-2c, -2d, -2e, or -2f (**A**). The more highly magnified images were taken from the samples of (**A**,**B**). At 48 hpt, the cells were fixed and stained with primary antibodies (anti-HA tag Ab for MERS-2c, -2d, -2e, and -2g, anti-MERS-S, or anti-MV F Abs). Nuclei were stained with DAPI.

We next analyzed HAHPs within the S gene region of MERS-CoV (GenBank accession: NC_019843). In the +2 frame of the MERS-CoV S gene, there were nine ORFs, which may encode peptides longer than 20 amino acids (Figure 2E). Although all these peptides contained many hydrophobic amino acids, analysis by the SOSUI program [25,26] predicted that six of the peptides have transmembrane hydrophobic alpha-helix regions. In this study, these were referred to as MERS-2c, -2d, -2e, -2f, -2g, and -2h (Figure 2E, Dataset S7). When these HAHPs were expressed in cells, MERS-CoV S-mediated syncytium formation was suppressed by MERS-2c, -2d, -2e, and -2f (Figure 5A). Among them, MERS-2c, -2e, and -2f, but not MERS-2d, co-localized with the MERS-CoV S protein (Figure 5B). The inhibition of syncytium formation was also specific to the S protein of MERS-CoV because the syncytium formation by measles virus glycoproteins was not affected by these HAHPs (Figure 5A).

### 2.4. Cross-Inhibition of Membrane Fusion by SARS-CoV-2 and MERS-CoV HAHPs

**Figure 6:**
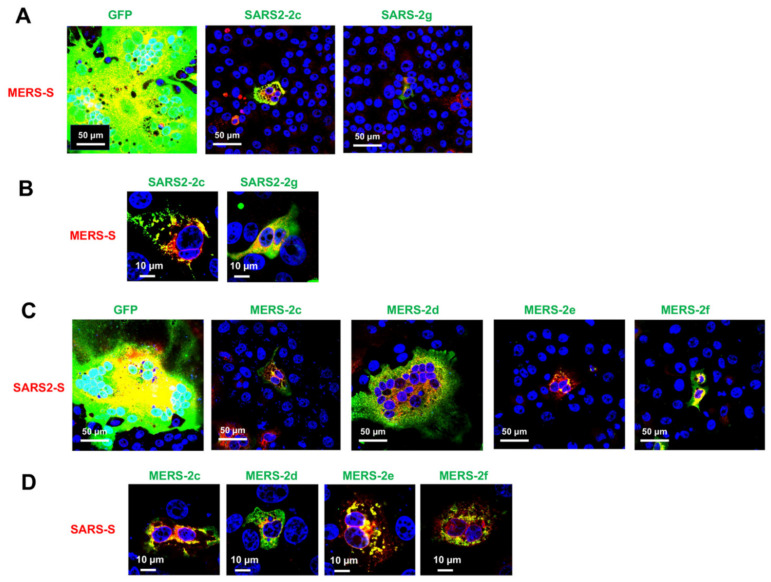
Cross membrane fusion inhibition by SARS-CoV-2 and MERS-CoV hydrophobic alpha-helical peptides (HAHPs). VeroE6/TMPRSS2 cells were co-transfected with expression plasmids for a combination of MERS-S plus GFP, SARS2-2c, or -2g (**A**,**B**), SARS-CoV-2 (SARS2)-S plus GFP, MERS-2c, -2d, -2e, or -2f (**C**,**D**). At 48 hpt, the cells were fixed and stained with primary antibodies as described in legends of Figure 4 and Figure 5. Nuclei were stained with DAPI.

The S proteins of coronaviruses share a high degree of structural and functional similarity. Therefore, we analyzed whether HAHPs of SARS-CoV-2 could inhibit the syncytium formation of MERS-CoV S protein and vice versa. As observed with the SARS-CoV-2 S protein, MERS-CoV S-mediated syncytium formation was suppressed by SARS2-2c and -2g (Figure 6A). Furthermore, as observed with the SARS2-S protein, SARS2-2c almost perfectly co-localized with the MERS-CoV2 S protein, while SARS2-2g partially co-localized with the MERS-CoV S protein (Figure 6B). Similar results were obtained with MERS-HAHPs and SARS2-S protein (Figure 6C,D). The SARS-CoV-2 S-mediated syncytium formation was suppressed by MERS-2c, -2e, and -2f, which exhibited co-localization with the SARS2-S protein (Figure 6C,D). Therefore, these HAHPs might have an inhibitory effect on different coronaviruses.

### 2.5. Viral RNA Synthesis Inhibition by HAHPs

**Figure 7:**
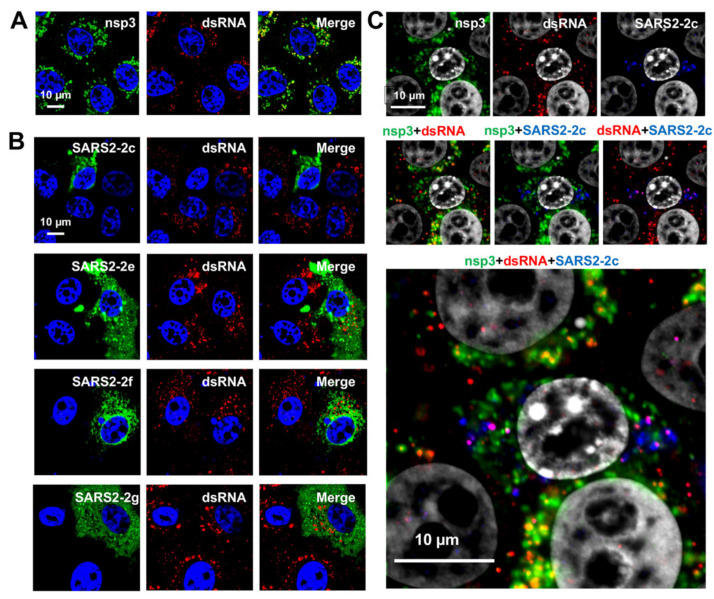
Viral RNA synthesis inhibition by SARS-CoV-2-2c. VeroE6/Rep3 cells were mock-transfected or singly transfected with a plasmid expressing SARS-CoV-2 (SARS2)-2c, -2e, -2f, or -2g. At 48 hpt, the cells were fixed and co-stained with anti-nsp3 and anti-dsRNA Abs for mock-transfected cells (**A**), anti-HA Ab and anti-dsRNA for SARS2-2c-, -2e-, -2f-, or -2g-transfected cells (**B**) and anti-nsp3, anti-dsRNA, and anti-HA Abs for SARS2-2c-transfected cells (**C**). Nuclei were stained with DAPI.

Coronavirus RNA synthesis occurs in specialized double-membrane vesicles (DMVs) induced by coronavirus infection [29,30]. The nonstructural proteins, nsp3 and nsp4, are critical components of coronaviral DMVs [29,30]. We considered that HAHPs, owing to having the properties of transmembrane hydrophobic peptides, could be incorporated into the DMV membrane or be a DMV component; therefore, we next analyzed the effect of HAHPs on viral RNA synthesis. This analysis was performed using a stable SARS-CoV-2 replicon cell line (VeroE6/Rep3), in which viral RNA synthesis and protein production occur continuously [31]. Double-stranded (ds)RNA is an intermediate product of coronavirus replication and is not detected in cells under normal (uninfected by virus) conditions [30]. We confirmed that dsRNA was detected in the cytoplasm in the form of dots, which co-localized with nsp3 (Figure 7A). SARS-CoV-2 HAHPs (SARS2-2c, -2e, -2f, and -2g) and MERS-CoV HAHPs (MERS-2c, -2e, -2f, and -2g) were expressed in VerE6/Rep3 cells. The dsRNA signal was specifically reduced in SARS2-2c-expressing cells, indicating that SARS2-2c affects viral RNA synthesis (Figure 7B). Next, triple staining with nsp3, dsRNA, and SARS2-2c was performed; in SARS2-2c-expressing cells, the remaining dsRNA signals were no longer co-localized with nsp3 (Figure 7C). They preferentially co-localized with SARS2-2c (Figure 7C). Other HAHPs did not co-localize with dsRNA (Figure 7B, Supplementary Figure S2) or nsp3 (Supplementary Figure S3). These data suggest that SARS2-2c targets DMVs and inhibits viral RNA synthesis by disrupting the nsp3-containing DMVs.

## 3. Discussion

In this study, we showed a unique property of the coronavirus genome. The presence of hydrophobic alpha-helical short peptides (referred to as HAHPs) was deduced in the coronavirus genomes in a number and frequency that cannot be considered coincidental. Previous studies [32,33,34,35] have reported an extreme codon usage bias (predominant selection of U for the third position of the codon) in coronavirus genomes and suggested that potential reasons for this bias include to help evade innate immunity by suppressing the CpG dinucleotide content and to contribute to the high gene expression. The third nucleotide in the +1 frame corresponds to the second nucleotide in the +2 frame, and all codons with U as the second nucleotide encode hydrophobic amino acids. Thus, this study suggests another potential reason for the extreme codon bias of the coronavirus genome. The third nucleotide in the frame, which encodes the major coronaviral proteins, is preferentially selected for U to encode a hydrophobic amino acid in the overlapping frame (if the frame encoding a major coronaviral protein is referred to as the +1 frame, the frame coding many hydrophobic amino acids is the +2 frame).

The most important question is whether such HAHPs are expressed in coronavirus-infected cells. Because the initiation codon of HAHP ORFs is located downstream of the initiation codon of the major coronavirus proteins in canonical sgRNAs, HAHPs are unlikely to be translated from these canonical sgRNAs. However, the internal entry of ribosomes or a ribosome leaky scanning may be used to translate HAHPs, as has been observed for specific coronavirus accessory proteins [36,37,38,39]. Another plausible possibility is translation from non-canonical sgRNAs. Recent studies have shown that non-canonical sgRNAs are synthesized at one-third to one-half the total amount of canonical sgRNAs [16,18,19], and many HAHP ORFs are located in the first position of certain non-canonical sgRNAs [14]. The expression of one HAHP has already been validated. HLA-I immunopeptidome detected peptides processed from internal out-of-frame ORFs in the S and N protein-coding regions [40]; they are termed S.iORF1 (or ORF2b [41]) and ORF9b, respectively [40]. S.iORF1 has all the characteristics of a HAHP that we describe in this paper. S.iORF1 is a 39-amino-acid peptide encoded in a reading frame with overlapping that of the S protein. We confirmed that S.iORF1 has a transmembrane hydrophobic alpha-helix structure, as predicted by SOSUI [25,26]. As with other HAHPs, the expression mechanism of S.iORF1 has not yet been resolved, but the ORF of S.iORF1 is also located at the first position of certain non-canonical sgRNAs [14].

Although we have not been able to confirm the expression of HAHPs in infected cells or infected individuals, we consider the finding that these HAHPs specifically affected critical steps in coronavirus propagation to be significant. The membrane fusion inhibitory activity of several SARS-CoV-2 or MERS-CoV HAHPs was shown to be specific to coronaviruses but may act broadly against coronaviruses. Considering the short length and the nature of the transmembrane hydrophobic alpha-helix region of these HAHPs, which inhibited S protein membrane fusion activity, the HAHPs may form a specific interaction with the transmembrane region or somewhere in the vicinity of the transmembrane region of the coronavirus S protein. The coronavirus S proteins have a highly conserved tryptophan-rich region in and near the transmembrane region that contacts the outer membrane of the lipid bilayer [42,43]. This region is essential in membrane fusion activity [42,43]. Therefore, we anticipate that this region is a potential target for these fusion-inhibitory HAHPs.

Although it is unknown whether all HAHPs are expressed and functional, coronaviruses encode many HAHPs throughout their ~30-kb long genomes. Many HAHPs are predicted to have a hydrophobic alpha-helix region, but there is a great deal of variation in HAHP amino acid sequences, HAHP length, the position of alpha-helices within HAHPs, and HAHP coding position within the genome. Coronaviruses may have diverse effects on host cells by combining many different HAHPs. However, conducting a functional analysis of HAHPs is expected to be more difficult compared with simply assessing single proteins. We speculate that involvement in viral RNA synthesis is one of the essential functions of HAHPs. Indeed, we observed that one HAHP (SARS2-2c) accumulated explicitly at the site of coronavirus RNA synthesis (possibly by incorporation into the DMV membranes) and inhibited RNA synthesis, although the details of this remain to be studied further. Because the assay conditions differ greatly from natural infection conditions under which each HAHP may be expressed in small amounts, we consider the assay results as evidence that HAHPs are a factor involved in RNA synthesis rather than that this HAHP has an RNA synthesis inhibitory activity.

Regarding the inhibition of S-protein-mediated membrane fusion by SARS2-2c, this study did not yet show how the fusion-inhibitory activity of SARS2c benefits SARS-CoV-2 replication in infected cells. Our preliminary experiments showed that the cell surface expression of S proteins was reduced in cells co-expressed with SARS2-2c. Coronavirus buds into the ER-Golgi intermediate compartment (ERGIC) to form viral particles, which are released extracellularly [44]. To achieve this, the S protein has an ER-retention signal in the cytoplasmic tail [45]. Thus, expression of the S protein at the cell surface is not critical for the coronavirus assembly. Rather, expression of the S protein on the cell surface is detrimental to the virus because it leads to immune recognition by the host. Thus, it is possible that SARS2-2c is involved in the reduced expression of S proteins at the cell surface and efficient particle formation at ERGIC, but further studies, including animal studies, will be needed to reach this conclusion. It is also possible that SRAS2-2c degraded the S protein. Indeed, recent studies have shown that artificial peptides that bind to the S protein of SARS-CoV-2 induce intracellular degradation of the S protein via the proteasome pathway [46]. If so, certain HAHPs could be important tools in the development of new drugs to inhibit SARS-CoV-2 infection.

In conclusion, this study found that coronaviruses potentially encode many hydrophobic short peptides with alpha-helix structures throughout the entire virus genome. We also demonstrated that some of these peptides have functions that specifically affect coronaviral membrane fusion and RNA synthesis. This study will spark a new discussion about our understanding of coronaviruses and will stimulate further research in this area.

## 4. Materials and Methods

### 4.1. Isolation and Sequencing of HCoVs

Nasopharyngeal swab specimens were collected from pediatric inpatients in Japan between 2018 and 2021, and those that were found to be HCoV positive by multiplex real-time PCR assays were used for virus isolation using an air–liquid interface culture of human bronchial tracheal epithelial cells as described previously [47]. The whole viral genome sequence analysis was performed as described previously [48]. The resulting sequence data were deposited to DDBJ/GenBank database. The accession numbers of HCoV-229E (Fukushima_H829_2020), -NL63 (Fukushima_H219_2018), -OC43 (Fukushima_H148_2018), and -HKU1 (Tokyo/SGH-18/2016) are LC654445.1, LC654455.1, LC654454.1, and LC315651.2, respectively. Human subjects were enrolled after approval from the ethics committee of the National Institute of Infectious Diseases, Japan (approval numbers 1001 and 1087).

### 4.2. Cells

VeroE6/TMPRSS2, Vero/hSLAM, HeLa/TMPRSS2 cells, and stable SARS-CoV-2 replicon VeroE6/Rep3 cells were reported previously [31,49,50,51] and maintained in Dulbecco Modified Eagle’s Medium (DMEM) containing 10% fetal bovine serum (FBS), 1% penicillin-streptomycin, and 0.5 mg/mL G418 (Sigma, Darmstadt, Germany).

### 4.3. Plasmids

The codon-optimized S protein expression plasmid (VG40589-UT) of the Wuhan strain of SARS-CoV-2 was purchased from Sino Biological, and the open reading frame of the S protein was cloned into a pCAGGS vector [52]. The cDNAs encoding the S protein of the Alpha (hCoV-19/Japan/QHN001/2020), Beta (hCoV-19/Japan/TY8-612/2021), Gamma (hCoV-19/Japan/TY7-501/2021), Delta (hCoV-19/Japan/TY11-927-P1/2021), and Omicron (hCoV-19/Japan/TY38-873P0/2021) variants were obtained from each viral RNA by conducting a reverse transcription reaction and were then cloned into pCAGGS vectors [52]. The HA epitope tag was added to the N-termini of all HAHPs (2c, 2d, 2e, 2f, and 2g for SARS-CoV-2 and 2c, 2d, 2e, 2f, 2g, 2h for MERS-EMC), which overlap within the S genes of the Delta strain of SARS-CoV-2 and the MERS-EMC strain, respectively, after which they were also cloned into pCAGGS vectors [52]. The cDNAs encoding the S protein of MERS-CoV EMC/2012 strain (GenBank accession: NC_019843), the hemagglutinin (HA) protein of influenza A/Puerto Rico/8/34 (PR8) strain with H141T and E142Q mutations [53], the fusion (F) and hemagglutinin-neuraminidase (HN) of SeV Z strain [54], and green fluorescent protein (GFP) were similarly cloned into pCAGGS vectors [52]. The measles virus (MV) F and hemagglutinin (H) expression plasmids were described previously [55].

### 4.4. Indirect Immunofluorescence Assay

VeroE6/TMPRSS2 cells, Vero/SLAM cells, HeLa/TMPRSS2 cells, and stable SARS-CoV-2 replicon VeroE6/Rep3 cells were seeded into six-well plates (1 × 106 cells/well), and the transfection of these cells with protein expression plasmids was carried out using Lipofectamine LTX (Invitrogen, Carlsbad, CA) or TransIT-LT1 reagent (Mirus Bio, Madison, WI). At 48 h post-transfection (hpt), the cells were fixed with 10% buffered formalin solution and then permeabilized with 0.5% Triton-X 100. After blocking, the cells were incubated with primary antibodies (Abs) and subsequently with secondary Abs conjugated with Alexa Fluor 488, 594, or 647 (Molecular Probes, Eugene, OR). The following Abs were used as primary Abs: rabbit anti-SARS-CoV-2 S protein polyclonal Ab (pAb) (Proteintech, Rosemont, IL), rabbit anti-MERS-CoV S protein pAb (Sino Biological, Beijing, China), mouse anti-HA tag monoclonal Ab (mAb) (Proteintech), rabbit anti-HA tag pAb (MBL), rat anti-HA tag mAb (Proteintech), mouse anti-HA mAb12-1G6 (for the detection of PR8 HA) [53], rabbit anti-SeV F pAb [56], mouse anti-MV F mAb [55], anti-dsRNA mAb J2 (SCICONS, Susteren, The Netherlands), and rabbit anti-SARS Nsp3 pAb (Abcam, Cambridge, UK). Nuclear staining was carried out with 4′,6′-diamidino-2-phenylindole (DAPI). The cells were observed with a laser scanning confocal microscope (FV3000, Olympus, Tokyo, Japan).

### 4.5. Protein Structure Prediction

The transmembrane helices in peptides and peptide structures were predicted by SOSUI ver. 1.11 “Available online: https://harrier.nagahama-i-bio.ac.jp/sosui/ (accessed on 23 June 2022)” [25,26], by TMHMM 2.0 “Available online: https://services.healthtech.dtu.dk/service.php?TMHMM-2.0 (accessed on 23 June 2022)”, and by Alphafold2 [28]. These structures were visualized using PyMOL (Molecular Graphics System, Version 2.5 Schrödinger, LLC.).

## Data Availability

Not applicable.

## Supplementary Materials

The following supporting information can be downloaded at: https://www.mdpi.com/article/10.3390/pathogens11080877/s1, Figure S1: Broad membrane fusion inhibitory activity in SARS-CoV-2 variants by SARS-CoV-2 2c.; Figure S2: No viral RNA synthesis inhibition by MERS-CoV-2 hydrophobic alpha-helical peptides (HAHPs).; Figure S3: Intracellular distribution of SARS-CoV-2 and MERS-CoV-2 hydrophobic alpha-helical peptides (HAHPs) and nsp3.; Dataset S1: Complete ORF list of HCoV-229E, deduced amino acid sequence of peptides, and predicted transmembrane hydrophobic helices.; Dataset S2: Complete ORF list of HCoV-NL63, deduced amino acid sequence of peptides, and predicted transmembrane hydrophobic helices.; Dataset S3: Complete ORF list of HCoV-OC43, deduced amino acid sequence of peptides, and predicted transmembrane hydrophobic helices.; Dataset S4: Complete ORF list of HCoV-HKU1, deduced amino acid sequence of peptides, and predicted transmembrane hydrophobic helices.; Dataset S5: Complete ORF list of SARS-CoV-2, deduced amino acid sequence of peptides, and predicted transmembrane hydrophobic helices.; Dataset S6: Complete ORF list of SARS-CoV, deduced amino acid sequence of peptides, and predicted transmembrane hydrophobic helices.; Dataset S7: Complete ORF list of MERS-CoV, deduced amino acid sequence of peptides, and predicted transmembrane hydrophobic helices.
