# Supplementary figures and images for: Hydrophobic Alpha-Helical Short Peptides in Overlapping Reading Frames of the Coronavirus Genome
Source: Pathogens. 2022 Aug 3;11(8):877. doi: 10.3390/pathogens11080877 (PMC9415614; doi:10.3390/pathogens11080877)

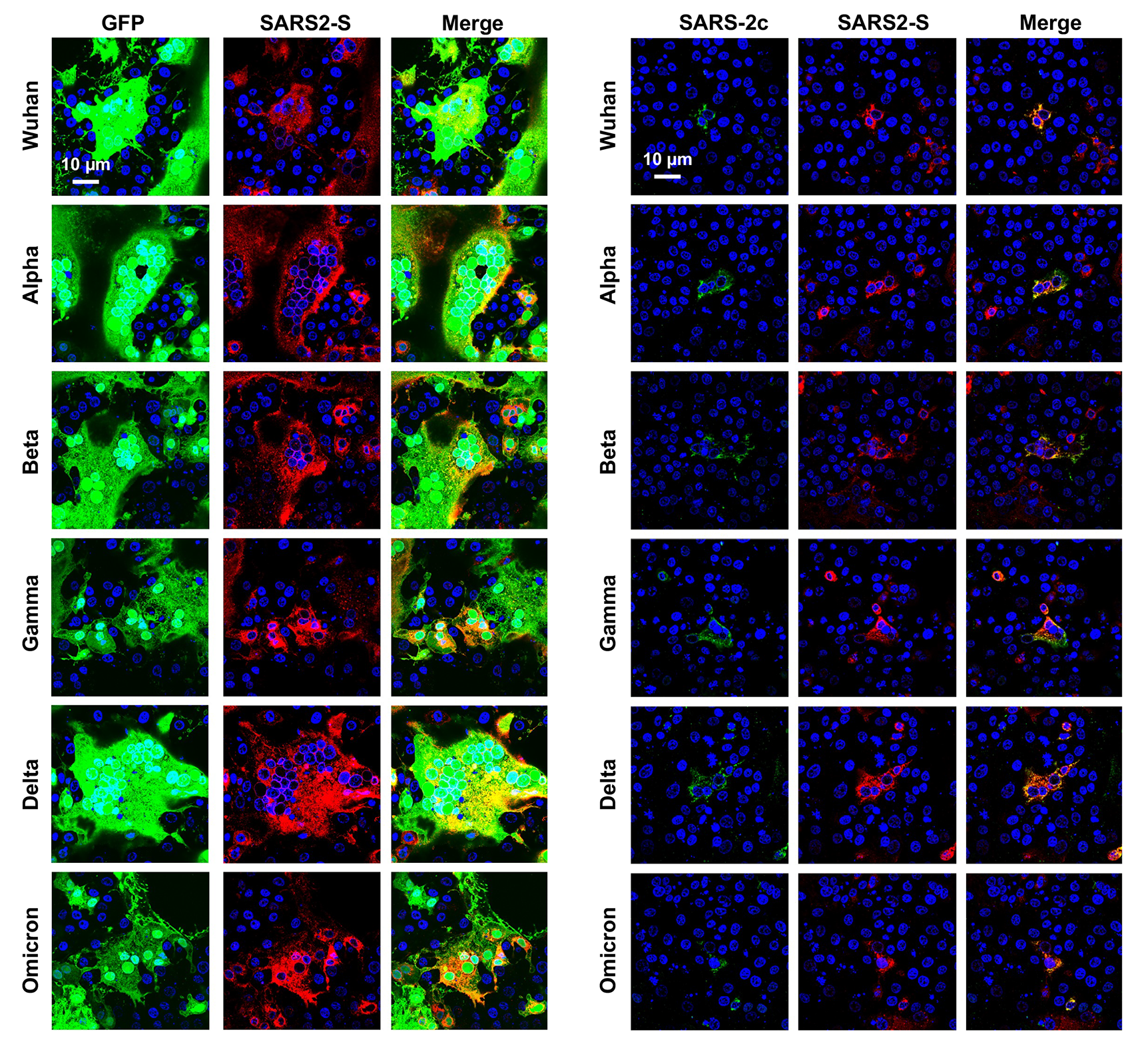

Supplement: Supplementary file 1 [file pathogens-11-00877-s001.zip › pathogens-1807283-supplementary/figure S1.tif]

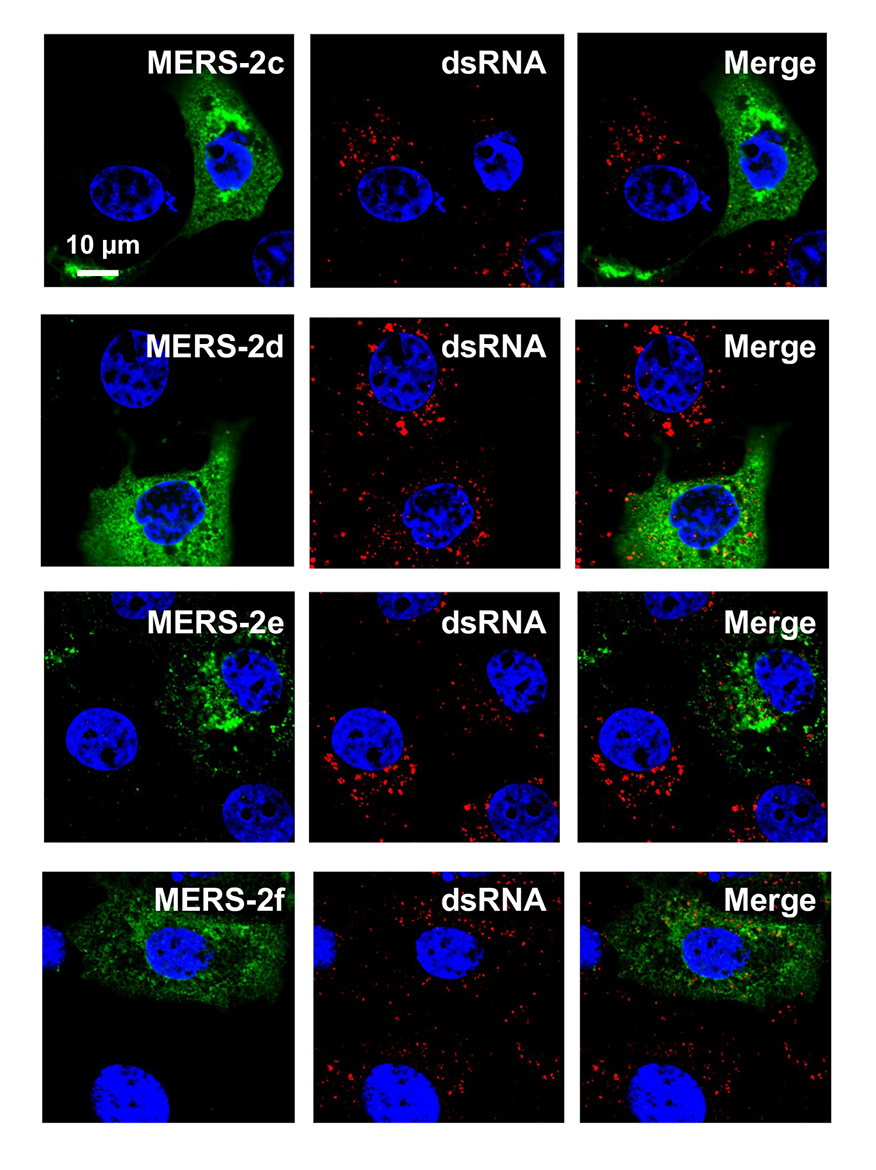

Supplement: Supplementary file 1 [file pathogens-11-00877-s001.zip › pathogens-1807283-supplementary/figure S2.tif]

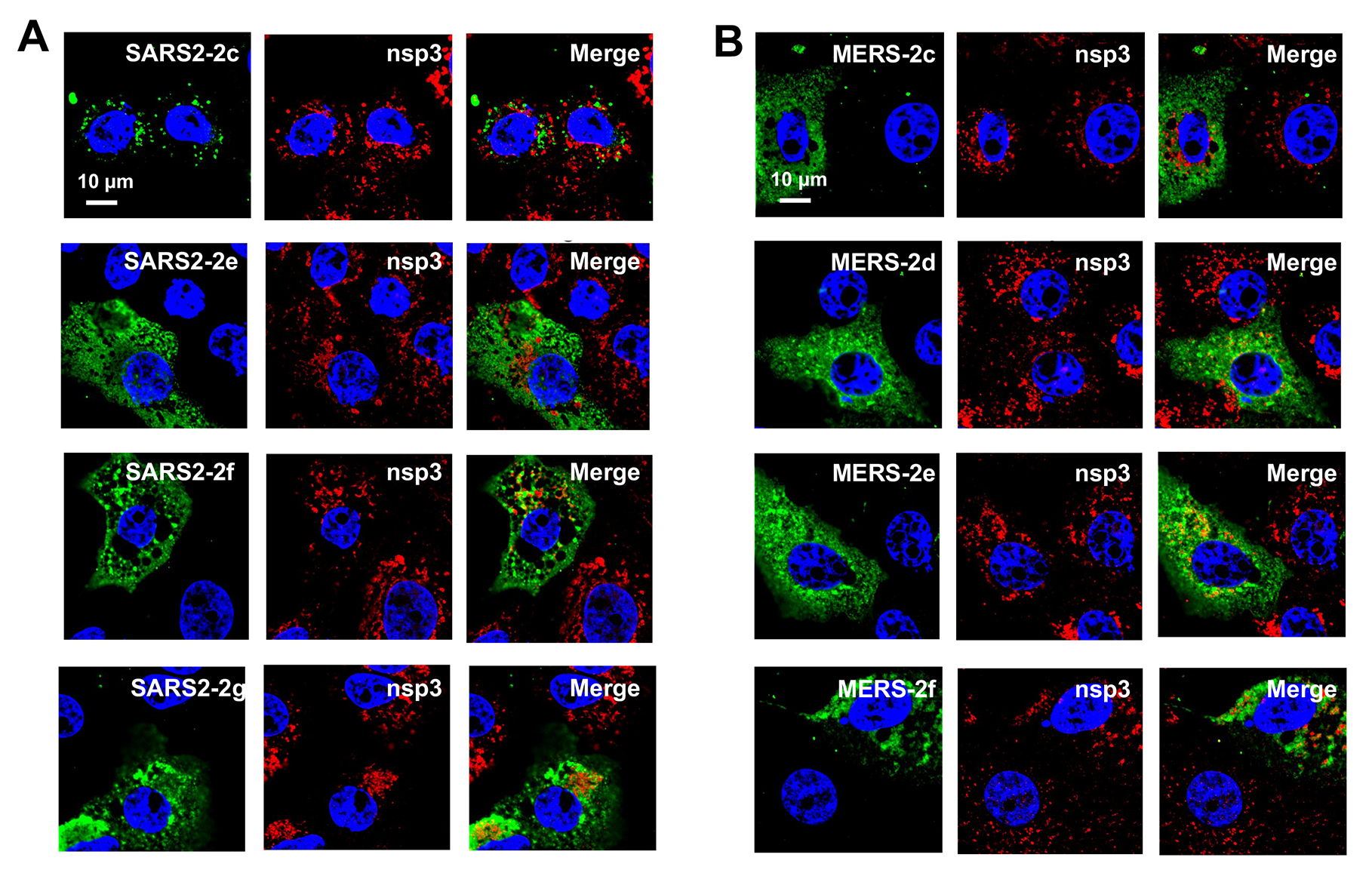

Supplement: Supplementary file 1 [file pathogens-11-00877-s001.zip › pathogens-1807283-supplementary/figure S3.tif]
